# Evaluation of Adefovir PBPK Model to Assess Biomarker‐Informed OAT1 Drug–Drug Interaction and Effect of Chronic Kidney Disease

**DOI:** 10.1002/psp4.70010

**Published:** 2025-03-03

**Authors:** Shawn Pei Feng Tan, Huaying Wu, Amin Rostami‐Hodjegan, Daniel Scotcher, Aleksandra Galetin

**Affiliations:** ^1^ Centre for Applied Pharmacokinetic Research, School of Health Sciences University of Manchester Manchester UK; ^2^ Certara Predictive Technologies Sheffield UK

**Keywords:** chronic kidney disease, endogenous biomarker, organic anion transporters, physiologically‐based pharmacokinetic modeling

## Abstract

Evaluation of transporter‐mediated drug–drug interactions (DDI) with endogenous biomarkers, coupled with physiologically‐based pharmacokinetic modeling (PBPK) is envisioned to replace or reduce dedicated DDI clinical trials with clinical probe substrates. The current study developed a PBPK model of adefovir (OAT1 clinical probe) by incorporating experimental measurements of adefovir passive diffusion and OAT1‐mediated transport in a mechanistic kidney model for in vitro*–*in vivo extrapolation of its secretion and renal clearance. The adefovir model was verified with clinical data from nine studies (188 subjects) investigating a range of adefovir intravenous and adefovir‐dipivoxil oral doses in White, Japanese, and Chinese populations with healthy renal function. A previously verified probenecid model, incorporating in vivo OAT1/3 inhibitory constant estimated using OAT1/3 endogenous biomarker (4‐pyridoxic acid) clinical data, was utilized. The ratio of adefovir maximal plasma concentrations (C_max_R) and area under the curve (AUCR) after mid‐to‐high single oral doses of probenecid was predicted within the stringent Guest criterion. With the lowest probenecid dose (0.5 g), adefovir AUCR and C_max_R were slightly overpredicted but still within a 1.5‐fold error. Application of the adefovir model to patients with severe chronic kidney disease (CKD) accounted for our previously recommended additional 50% decrease in OAT1 activity beyond the decline of glomerular filtration rate. The model successfully predicted 3‐fold higher adefovir C_max_ and 6‐fold higher AUC in patients with severe CKD relative to healthy individuals. This study reinforces the role of PBPK modeling to predict transporter‐mediated DDI (coupled with biomarker‐informed approach to optimize in vivo inhibitory constant) and the effect of renal impairment on OAT1/3 drugs in lieu of clinical studies.

**Summary**

- What is the current knowledge on the topic?
  ○ Coupling PBPK modeling with endogenous biomarker data has the potential to inform transporter‐mediated drug–drug interactions (DDI) clinical studies.
- What question did this study address?
  ○ Can a biomarker‐informed PBPK modeling approach predict the renal organic anion transporter 1 (OAT1)‐mediated DDI between probenecid and adefovir? Would PBPK modeling predict the effect of chronic kidney disease (CKD) on adefovir pharmacokinetics and provide a preliminary assessment of DDI risk in CKD?
- What does this study add to our knowledge?
  ○ The first application of an OAT1/3 inhibitor model verified with endogenous biomarker (4‐pyridoxic acid) clinical data to predict the OAT1‐mediated DDI with clinical probe adefovir. Successful prediction of changes in adefovir pharmacokinetics in CKD reaffirms the need to reduce OAT1 activity beyond the decline of glomerular filtration rate in severe CKD PBPK models.
- How might this change drug discovery, development, and/or therapeutics?
  ○ PBPK modeling has the potential to inform dedicated DDI studies evaluating OAT1‐mediated inhibition (while leveraging on transporter biomarker clinical data) and reduce the need for clinical studies investigating the effect of renal impairment on OAT1‐renally eliminated drugs.

## 1 Introduction

The quantitative prediction of metabolic drug–drug interactions (DDI) is now a common practice in drug development and regulatory submissions [1, 2]. In contrast, the successful prediction of transporter‐mediated DDI is still challenging [3, 4]. This trend is often related to a disconnect between in vitro and in vivo inhibitory potency for a new chemical entity (NCE) against a transporter of interest [5]. Moreover, it is recognized that static DDI assessment thresholds recommended by regulatory guidances [6, 7, 8] are conservative, leading to false positives and unnecessary DDI clinical studies with the transporter clinical probe [9, 10]. Monitoring endogenous biomarkers of transporters has been increasingly used for in vivo assessment of potential transporter‐mediated DDI [11, 12, 13] and has been highlighted as an acceptable approach for DDI risk assessment in the International Council for Harmonization M12 guidance [6]. Measuring transporter biomarkers during phase I trials aims to guide the decision whether to prioritize, delay, or replace the need for additional DDI clinical studies [5, 14]. The International Transporter Consortium (ITC) has recently classified transporter endogenous biomarkers into several tiers based on the existing evidence, with a tier 1 biomarker (coproporphyrin‐I) considered fully validated and recommended to be measured in Phase I studies for clinical DDI risk assessment [5]. In addition, the integration of biomarker clinical data with modeling and simulation (physiologically‐based pharmacokinetic (PBPK) or population pharmacokinetic (PopPK) modeling) enables estimation of the in vivo inhibitory constant (K_i_) of the NCE for a transporter [13, 15, 16]. Subsequent use of the endogenous biomarker‐informed inhibitor model for prospective prediction of the DDI between NCE and transporter clinical probe has been envisioned to replace the need for some dedicated clinical DDI studies, as recently demonstrated for coproporphyrin‐I as a biomarker for organic anion transporting polypeptide (OATP1B1/3) [13].

In addition to OATP1B1/3, numerous biomarkers have been identified for renal transporters such as organic anion transporters (OAT1/3), organic cation transporter, and multidrug and toxin extrusion protein 1/2‐K thus far [5, 14]. The OAT1/3 are responsible for the active renal secretion of drugs such as beta‐lactam antibiotics, antivirals, and diuretics [14]. In vitro evaluation of OAT1/3 inhibition by an investigational drug is recommended by regulatory agencies [6]. 4‐pyridoxic acid (PDA) was previously identified as a sensitive and specific endogenous biomarker of OAT1/3 [17, 18, 19, 20]. Several studies have demonstrated the sensitivity of PDA plasma and urine levels to probenecid inhibition (strong OAT1/3 clinical inhibitor) [17, 19, 20], whereas no interaction was observed with inhibitors of other hepatic and renal transporters [20]. Due to a lack of clinical DDI data with weak and moderate OAT1/3 inhibitors and any model‐based evaluation of a DDI with OAT1/3 clinical probes, PDA is currently classified as a tier 2 biomarker [5], and hence not considered a fully validated biomarker. We previously developed and verified a PBPK model for PDA, utilizing clinical data with and without probenecid interaction, and from patients with CKD [21]. In that study, probenecid OAT1/3 K_i_ that was previously estimated using PopPK modeling [15] of PDA clinical data was further verified against an independent PDA‐probenecid clinical dataset [17]. However, further validation of PDA as an OAT1/3 biomarker is needed. To address this, the current study applied the biomarker‐informed probenecid PBPK model to predict the extent of DDI with a selected OAT1 clinical probe. Considering uncertainty in the individual contribution of OAT1/3 for ciprofloxacin and furosemide and the availability of clinical data, our study focused on adefovir.

Adefovir is an OAT1‐specific [22] clinical probe recommended for the investigation of OAT1 inhibition in clinical studies [23, 24]. Adefovir is the active form of the orally administered prodrug, adefovir‐dipivoxil, and is almost entirely renally eliminated from the body [25]. Due to its high fraction unbound in plasma (f_u,*p*_ = 0.96 [26]), adefovir renal clearance occurs via both passive glomerular filtration and OAT1‐mediated active tubular secretion, with renal secretion contributing approximately 60% to its renal clearance [25]. In patients with chronic kidney disease (CKD), the pharmacokinetics of adefovir are considerably altered (6.2‐fold increase in AUC in severe CKD), leading to clinical dose reductions [26]. Analyses by our group [27] and others [28, 29] have highlighted that OAT1/3 activity decreases faster than the decline of glomerular filtration rate (GFR) in severe stages of CKD.

In this study, a PBPK model for adefovir was developed using experimental measurements of OAT1‐mediated adefovir active transport and passive diffusion across the renal proximal tubular cell. Verification of the adefovir model was performed using clinical pharmacokinetic data after intravenous and oral administration of adefovir‐dipivoxil (adefovir prodrug) in White, Japanese, and Chinese subjects. The application of the previously verified biomarker‐informed probenecid PBPK model to predict DDI with adefovir across a threefold range of probenecid doses was performed. Additional adefovir model verification was done against the reported clinical data from patients with mild, moderate, and severe CKD, with the aim of validating our previously proposed PBPK modeling framework in CKD populations.

## 2 Methods

### 2.1 Adefovir Clinical Data

**FIGURE 1:**
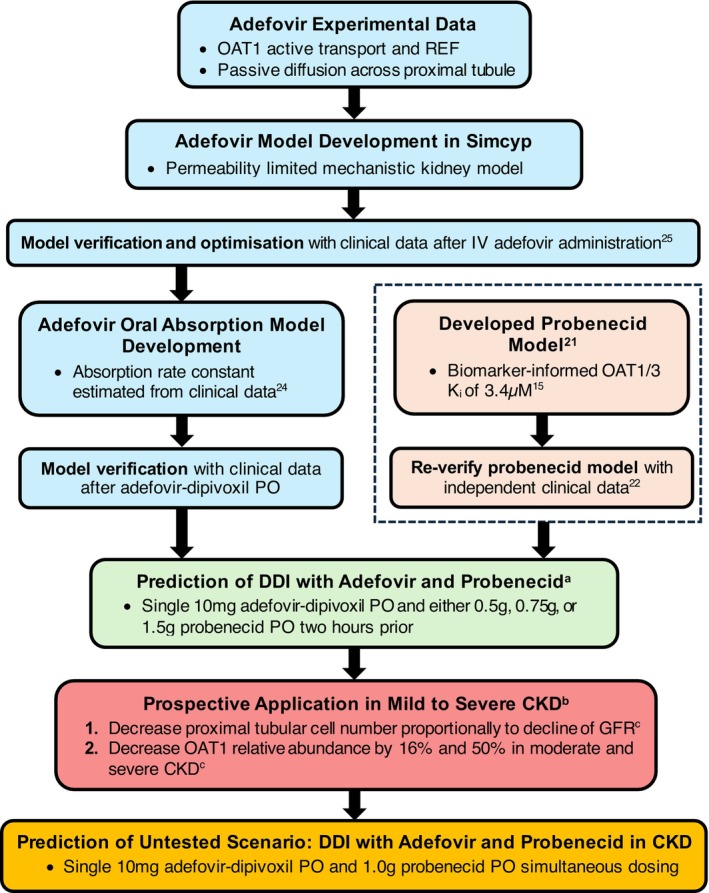
Adefovir model development workflow for healthy populations, drug–drug interaction (DDI) prediction with probenecid, and application in renal impairment. CKD, chronic kidney disease; GFR, glomerular filtration rate; IV, intravenous; K_i_, inhibitory constant; OAT1/3, organic anion transporter 1/3; PO, oral administration; REF, relative expression factor. ^a^Trial design, pharmacokinetic data, and demographic data obtained from Maeda et al. [22] (Table S3). ^b^Trial design, pharmacokinetic data, and demographic data in renal impaired subjects obtained from Hepsera Clinical Pharmacology and Biopharmaceutics Review [26] (Table S4). ^c^Changes implemented in Simcyp renal impairment population file (Table S1).

Data from nine clinical studies were collated for adefovir PBPK model development, verification, and CKD application. Seven clinical studies reported the pharmacokinetics of adefovir after single and/or multiple intravenous and oral doses of adefovir and adefovir‐dipivoxil, respectively (Figure 1, Table 1), whereas reported probenecid‐adefovir DDI data were obtained from a clinical study of six Japanese male subjects [22]. In the DDI study, the interaction between a single oral dose of 10 mg adefovir‐dipivoxil and 0.5, 0.75, and 1.5 g single oral doses of probenecid was investigated. Probenecid was given 2 h prior to the adefovir dose, with a washout period of 7 days between different probenecid doses investigated. Clinical plasma and urine data investigating the effect of CKD on adefovir pharmacokinetics were obtained from the Hepsera Clinical Pharmacology and Biopharmaceutics Review [26]. In total, this report contained pharmacokinetic data from 33 subjects with various extents of renal impairment, and all received adefovir‐dipivoxil 10 mg single oral dose. These subjects were stratified to CKD stages based on their creatinine clearance (CrCL) as follows: healthy: CrCL > 80 mL/min; mild CKD: CrCL 50–80 mL/min; moderate CKD: CrCL 30–50 mL/min; severe CKD: < 30 mL/min. Due to constraints of the reported clinical data, classification of CKD subjects differed slightly from recommendations in the latest FDA guidance [35].

**TABLE 1:** Adefovir clinical data used for PBPK model development, verification, drug–drug interaction prediction, and chronic kidney disease application.

	Reference	Drug administered	*N*	Dose	Number of doses
Model development and verification	Cundy et al. [25]	Adefovir	28	1.0 mg/kg and 3.0 mg/kg IV	Multiple once daily for 4 weeks
	Trueck et al. [24]	Adefovir‐DPV	24	10 mg PO	Single
	Kearney et al. [30]	Adefovir‐DPV	24	10 mg PO	Single
	Shida et al. [31]	Adefovir‐DPV	12	10 mg PO	Once daily at day 1 and day 4 to 8
	Sun et al. [32]	Adefovir‐DPV	19	10 mg, 20 mg and 40 mg PO	Single and multiple once daily for 7 days^a^
	Fok et al. [33]	Adefovir‐DPV	40	10 mg PO	Single
	Barditch‐Crovo et al. [34]	Adefovir‐DPV	27	125 mg, 250 mg and 500 mg PO	Multiple once daily for 14 days
DDI prediction	Maeda et al. [22]	Adefovir‐DPV, Probenecid	6^b^	Adefovir‐DPV: 10 mg PO Probenecid: 0.5 g, 0.75 g and 1.5 g PO	Single dose of adefovir‐DPV and probenecid^c^
CKD application	USFDA [26]	Adefovir‐DPV	33	10 mg PO	Single

Abbreviations: CKD, chronic kidney disease; DDI, drug–drug interaction; DPV, dipivoxil; IV, intravenous dose; PO, oral dose; USFDA, United States Food and Drug Administration.

^a^ Only multiple doses of 10 mg adefovir‐DPV were investigated.

^b^ Six subject were recruited across four trial phases in a crossover manner.

^c^ Single oral dose of probenecid was administered 2 h before adefovir‐DPV.

### 2.2 PBPK Model Development and Verification

Adefovir PBPK model was developed using Simcyp Simulator (Version 23, Certara, Sheffield, UK). Physicochemical properties of adefovir were collated from the literature (Table S1). Distribution was predicted using the full PBPK distribution model coupled with the Rodgers and Rowland method [36, 37]. The permeability‐limited mechanistic kidney model was used to predict the active renal secretion of adefovir via OAT1. Adefovir OAT1‐mediated intrinsic clearance (CL_int_) and passive diffusion clearance were obtained experimentally using conditionally immortalized proximal tubular epithelial cells overexpressing OAT1 (ciPTEC‐OAT1) [38]. Relative expression factor (REF) accounting for differences in OAT1 expression in vivo versus expression in vitro was used for in vitro‐to‐in vivo extrapolation (IVIVE) of OAT1‐mediated secretion clearance. REF value was based on reported OAT1 expression in human kidney cortex [39] and global proteomics measurement of OAT1 expression in ciPTEC‐OAT1 [38]. Further optimization of REF (two‐fold increase to OAT1 CL_int_) using clinical data after 1.0 mg/kg intravenous administration of adefovir [25] was necessary to recover its observed pharmacokinetic profile and renal clearance (CL_r_). It was assumed that the rate‐determining process [4] in adefovir renal secretion was basolateral uptake via OAT1 and a value of 1.0 μL/min/10^6^ PTC was used for multidrug resistance polypeptide 4 (MRP4) efflux of adefovir [40]. After initial verification of PBPK model with intravenous administration of 3.0 mg/kg adefovir, the PBPK model was expanded to include oral absorption. The 1st order absorption model was used with a single rate constant (k_a_) to describe both the intestinal absorption of adefovir‐dipivoxil and subsequent rapid hydrolysis to adefovir in the intestinal enterocytes and plasma. As it was not necessary for our model to define distinct molecular species (adefovir‐dipivoxil and adefovir), a 10 mg oral dose of adefovir‐dipivoxil was implemented as a 5.45 mg oral dose of adefovir based on the molecular weight of adefovir relative to adefovir‐dipivoxil. The k_a_ was optimized using the parameter estimation module and pharmacokinetic data after a single oral dose of adefovir‐dipivoxil [24].

Model verification was performed against clinical data from nine clinical studies (188 subjects) investigating a range of adefovir dosing in White, Japanese, and Chinese populations (Table 1). During verification, simulations were performed using the Simcyp Healthy‐Volunteers population file and a generic trial design of 10 trials of 20 subjects with an equal number of male/female subjects of age range between 20 to 50 years old. When verifying against clinical data from Japanese or Chinese subjects, the Sim‐Chinese and Sim‐Japanese population files provided in the Simcyp V23 were used. Genetic polymorphisms of OAT1 are infrequent and do not lead to changes in transporter activity [41]. Therefore, no inter‐ethnic differences in OAT1 activity were implemented in the simulations in Chinese or Japanese populations. Predictions of adefovir maximum plasma concentration (C_max_), area under the plasma concentration‐time profile (AUC) and CL_r_ were compared against the observed data. Predictions of adefovir pharmacokinetic parameters were deemed accurate when the ratio of predicted versus observed (R_pred/Obs_) fell within the 1.5‐fold error criterion (0.67 ≤ R_pred/Obs_ ≤ 1.5). Observed plasma concentration‐time profiles were digitized using WebPlotDigitizer (Version 4.4, Automeris LLC, Frisco, TX). All data analysis was performed using Microsoft Excel (Microsoft, Redmond, WA) and Graphpad Prism 10.2.2 (Graphpad Software, La Jolla, CA).

### 2.3 Prediction of DDI Between Adefovir and Probenecid

A previously developed probenecid PBPK model [21] was applied to predict the effect of probenecid OAT1/3 inhibition on adefovir pharmacokinetics (model input parameters found in Table S2). The in vivo OAT1/3 unbound K_i_ of 3.4 μM used in the probenecid PBPK model was estimated from clinical data investigating probenecid interaction with PDA (OAT1/3 endogenous biomarker) [15, 19].

Prediction of the DDI between adefovir and probenecid was performed by adopting the clinical trial design of the clinical study from Maeda et al. [22] (Figure 1, Table S3). Four trial phases were simulated, namely a control phase where a single oral dose of 10 mg adefovir‐dipivoxil was administered, followed by three DDI phases where a single oral dose of 0.5 g, 0.75 g, or 1.5 g probenecid was administered 2 h before adefovir‐dipivoxil. For each phase, 40 trials of 6 male subjects with an age range of 20–31 years were performed using the Sim‐Japanese population file. The Guest et al. [42] criterion was applied to evaluate predictions of changes to PK parameters (C_max_R, AUCR and CL_r_R) from the control phase due to probenecid inhibition. Observed C_max_R, AUCR, and CL_r_R were not reported in the clinical study and were therefore calculated using the reported mean pharmacokinetic parameters. The impact of dose timing on the magnitude of adefovir‐probenecid DDI was explored by PBPK modeling where additional simulations of adefovir‐dipivoxil and probenecid were administered simultaneously.

### 2.4 Prospective Application of Adefovir PBPK Model to Chronic Kidney Disease Populations

To predict the effect of mild, moderate, and severe CKD on adefovir pharmacokinetics, the Sim‐Renal Impaired_Mild, Sim‐Renal Impaired_Moderate, and Sim‐Renal Impaired_Severe population files were used. As OAT1 expression in the default Simcyp CKD population is unchanged relative to the healthy, we have made two modifications to the disease population files. Firstly, the absolute proximal tubular cell number of each CKD population file was reduced proportionally to the decrease in mean GFR, and based on the intact nephron hypothesis [43], as previously performed [21, 44, 45] (Figure 1, Table S1). Our previous analysis indicated that OAT1/3 activity declines faster than the decrease in GFR with CKD progression [27]. We recommended that additional reduction to OAT1/3 activity be considered in PBPK models if secretion clearance (CL_sec_) of an OAT1/3 substrate forms the majority of its CL_r_ (CL_sec_/CL_r_ ≥ 0.5). As adefovir met this criterion, its OAT1‐mediated CL_int_ was decreased by an additional 16% in moderate CKD and 50% in severe CKD [27] by adjusting the OAT1 relative abundance in the respective Simcyp population files from 1.0 (in healthy and mild CKD) to 0.84 (moderate CKD) and 0.50 (severe CKD) (Table S1).

Verification of the predicted adefovir pharmacokinetics in CKD was performed using clinical data from 33 individuals in healthy and mild to severe CKD [26]. Simulation design incorporated simulations of 30 trials with the respective number and the demographic data reported in the reference clinical study (Table S4). Plasma concentration‐time profiles were not reported in patients with CKD, and predictive performance was assessed against reported mean pharmacokinetic parameters (AUC, C_max_ and CL_r_) using a 1.5‐fold error criterion. Measured CL_r_ and corresponding GFR of individual subjects were reported and digitized using WebPlotDigitizer.

In addition to the prediction of adefovir PK, the model was used to explore untested clinical scenarios and to prospectively evaluate the significance of probenecid OAT1/3 inhibition on adefovir plasma pharmacokinetics and renal clearance in patients with CKD. The DDI between adefovir and probenecid in CKD was simulated using the respective verified PBPK models and modified Simcyp renal impaired population files using a generic trial design (Table S5). A single oral dose of 10 mg adefovir and 1.0 g probenecid given simultaneously was selected for simulations (both are within the recommended dose range in patients with CKD) [46, 47].

## 3 Results

### 3.1 Adefovir PBPK Model Development and Verification

**FIGURE 2:**
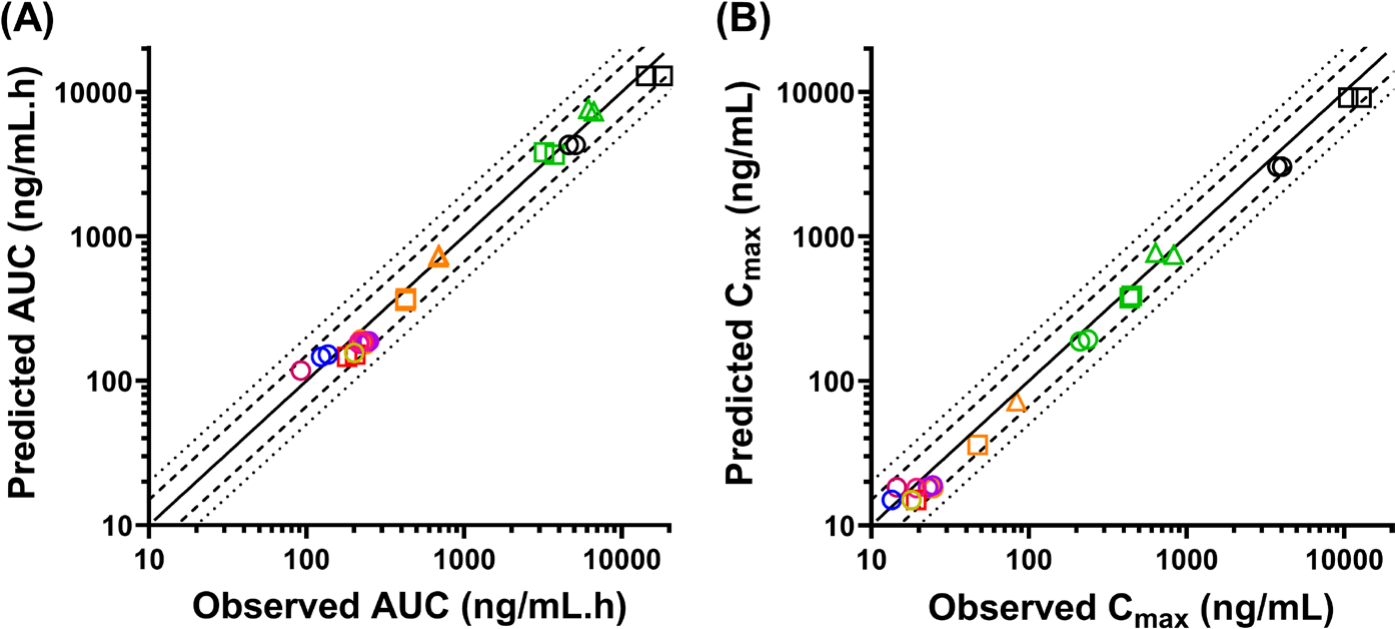
Verification of adefovir physiologically‐based pharmacokinetic model. Predicted versus observed adefovir area under the plasma concentration‐time profile (AUC, A) and maximum plasma concentration (C_max_, B). The solid, dashed and dotted lines represent the line of unity, 1.5‐fold and 2‐fold error criterion. Each colored symbol represents one of 29 AUC measurements and 22 C_max_ measurements obtained from nine clinical studies following administration of single and/or multiple 10 mg to 500 mg oral doses of adefovir‐dipivoxil, and 1.0 mg/kg and 3.0 mg/kg intravenous doses of adefovir in subjects with healthy renal function (details of studies in Table 1). All the observed AUC and C_max_ measurements were successfully predicted within 1.5‐fold of observed data. Only six clinical studies measured adefovir renal clearance (CL_r_), and seven of the eight reported CL_r_ measurements were predicted within 1.5‐fold. References: Cundy et al. [25](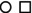), Trueck et al. [24](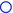), Kearney et al. [30] (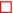), Shida et al. [31](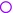), Fok et al. [33](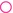), Sun et al. [32](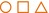), Barditch‐Crovo et al. [34] (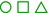), Maeda et al. [22](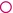) and US FDA [26](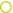).

Adefovir PBPK model was initially developed using experimental measurements of OAT1‐mediated active uptake of adefovir (CL_int_) and OAT1 REF [38]. This approach resulted in a 1.8‐fold overprediction of the observed CL_r_ and underprediction of the plasma concentration‐time profile after intravenous 1.0 mg/kg adefovir administration (Figure S1). Optimized OAT1 REF value of 2.0 was successfully verified against clinical data following intravenous administration of 3.0 mg/kg adefovir (Figure S2), with predicted C_max_, AUC, and CL_r_ within 1.5‐fold of the observed values. Predicted volume of distribution at steady‐state of 0.39 L/kg agreed with the observed value (0.42 L/kg). The k_a_ describing the rate of oral absorption and conversion of adefovir‐dipivoxil to adefovir was optimized using clinical data after a single oral dose of adefovir‐dipivoxil [24]. Fraction of adefovir‐dipivoxil absorbed and converted to adefovir was assumed to be 0.47 based on previous adefovir PBPK model [48] and was within the range of reported estimated bioavailability of 0.32 to 0.59 [26, 34]. In further verifications of the adefovir PBPK model, the AUC and C_max_ were successfully predicted within 1.5‐fold (Figure 2) of the observed data from eight clinical studies investigating a wide dose range (10 mg to 500 mg) of both single and multiple oral dosing of adefovir‐dipivoxil (Table 1).

### 3.2 Prediction of Adefovir and Probenecid DDI

**FIGURE 3:**
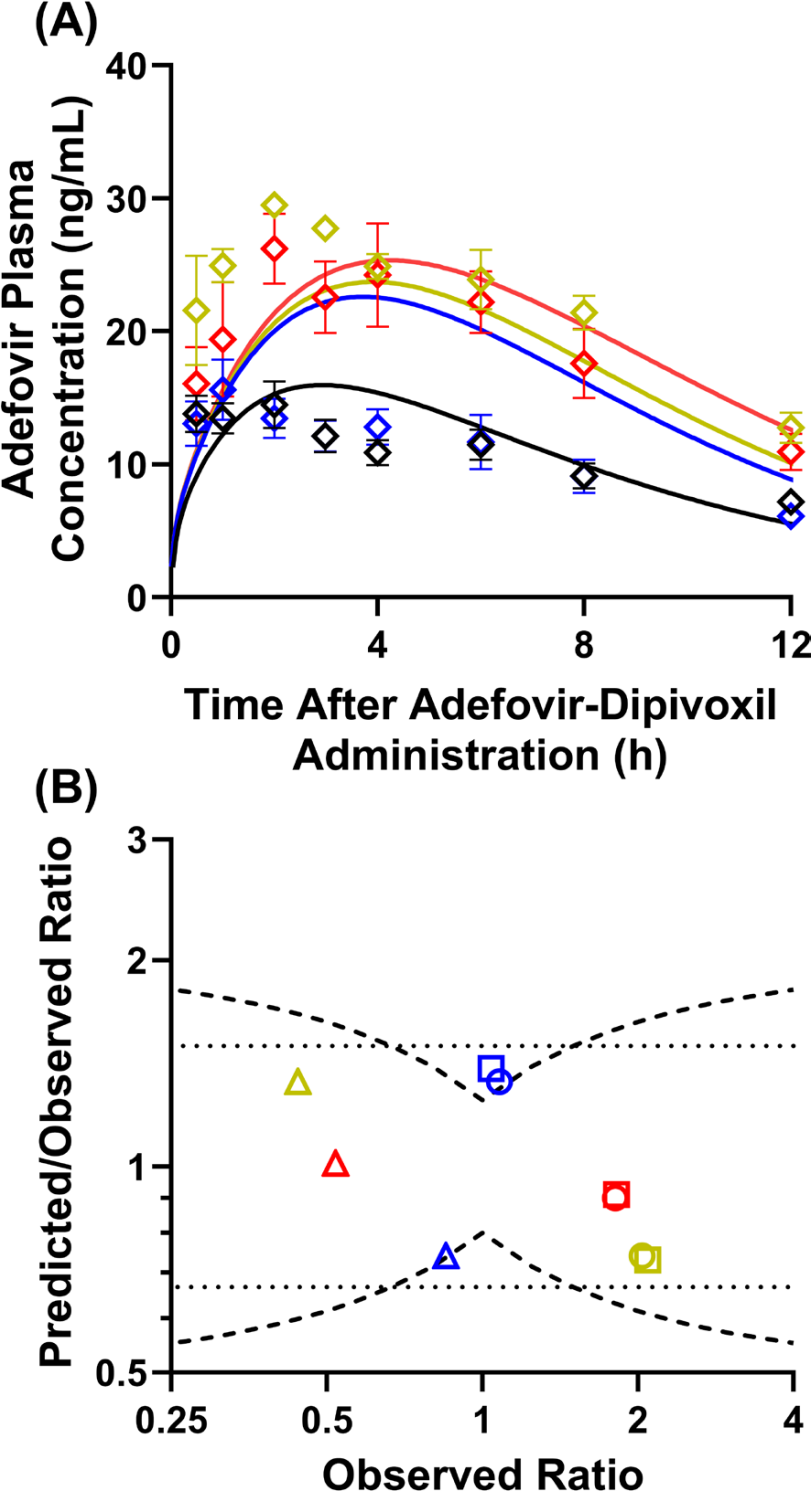
Drug–drug interaction (DDI) between adefovir‐dipivoxil and probenecid. (A) Predicted (solid line) versus observed adefovir plasma concentration‐time profile in the control phase (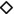) and during DDI with 0.5 g (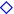), 0.75 g (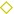) and 1.5 g (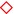) probenecid administered 2 h before 10 mg adefovir‐dipivoxil. The predicted mean and observed mean ± standard error adefovir plasma concentration are presented here. (B) Predicted/observed versus observed ratio (probenecid/control phase) of adefovir maximum plasma concentration (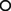), area under the curve (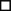) and renal clearance (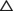). Blue, yellow and red symbols represent DDI with single oral dose of 0.5 g, 0.75 g and 1.5 g probenecid, respectively. Dashed and dotted lines represent the Guest criterion [42] and 1.5‐fold error criterion, respectively. Simulations were performed using the demographic data and trial design reported in the DDI clinical study (Table S3) [22].

Our previously verified probenecid PBPK model [21] was able to predict the observed plasma concentration‐time profile for probenecid after a single oral dose of 0.5 g, 0.75 g, and 1.5 g probenecid from the reference DDI study used in this work (Figure S3). Subsequent simulation of the adefovir‐probenecid DDI using verified PBPK models of adefovir and probenecid (incorporating a PDA‐informed in vivo OAT1/3 K_i_ of 3.4 μM) predicted well the extent of interaction with mid‐to‐high single oral doses of probenecid (0.75 g and 1.5 g) (Figure 3A). After a single oral dose of 0.5 g, 0.75 g, and 1.5 g probenecid, adefovir AUC was predicted to increase by 1.45‐, 1.53‐, and 1.65‐fold, respectively, in agreement with the reported data (1.04 to 1.82‐fold increase from the lowest to the highest probenecid dose) (Table 2). Adefovir DDI with the largest probenecid dose of 1.5 g had the best predictive performance, with C_max_R, AUCR, and CL_r_R predicted within Guest criterion and 1.25‐fold of the observed value (Figure 3B). However, interaction with the lowest probenecid dose (0.5 g) was slightly overpredicted compared with clinical data which did not observe any significant DDI (Figure 3A). Although the predicted AUCR, C_max_R, and CL_r_R at the lowest probenecid dose marginally exceeded the Guest criterion, they were still within 1.5‐fold of the observed values (Figure 3B). Simulations of simultaneous dosing of adefovir‐dipivoxil and probenecid predicted a comparable extent of DDI to the 2 h pre‐dosing of probenecid used in the clinical study (Figure S4).

**TABLE 2:** Summary of observed versus predicted changes to adefovir pharmacokinetic parameters in the presence and absence of probenecid interaction.

Pharmacokinetic Parameter	Control			0.5 g Probenecid			0.75 g Probenecid			1.5 g Probenecid		
	Pred.	Obs.	R_pred/obs_	Pred.	Obs.	R_pred/obs_	Pred.	Obs.	R_pred/obs_	Pred.	Obs.	R_pred/obs_
C_max_ (ng/mL)	16.2 ± 7.5	14.5 ± 1.8	1.12	23.0 ± 10.5	15.6 ± 2.3	1.48	24.2 ± 11.0	29.5 ± 0.7	0.82	25.8 ± 11.7	26.2 ± 2.6	0.99
AUC_0‐8h_ (ng/mL.h)	105 ± 49	92 ± 7.1	1.15	151 ± 69	95 ± 11	1.58	159 ± 72	191 ± 12	0.83	171 ± 77	167 ± 20	1.02
CL_r_ (L/h/kg)	0.24 ± 0.07	0.18 ± 0.02	1.35	0.15 ± 0.05	0.15 ± 0.02	0.99	0.14 ± 0.04	0.08 ± 0.01	1.77	0.12 ± 0.04	0.09 ± 0.01	1.34
C_max_R	N.A.	N.A.	N.A.	1.43 ± 0.17	1.08	1.33	1.51 ± 0.22	2.04	0.74	1.62 ± 0.29	1.81	0.90
AUC_0‐8h_R	N.A.	N.A.	N.A.	1.45 ± 0.19	1.04	1.39	1.53 ± 0.23	2.09	0.73	1.65 ± 0.31	1.82	0.91
CL_r_R	N.A.	N.A.	N.A.	0.63 ± 0.11	0.85	0.74	0.58 ± 0.12	0.44	1.33	0.52 ± 0.13	0.52	1.01

*Note:* Data are presented as mean ± standard deviation.

Abbreviations: AUC, Area under the plasma‐concentration time profile; CL_r_, renal clearance; C_max_, maximum plasma concentration; DDI, drug–drug interaction; N.A., Not applicable; R_pred/obs_, predicted/observed ratio.

### 3.3 Effect of Chronic Kidney Disease on Adefovir Pharmacokinetics

**FIGURE 4:**
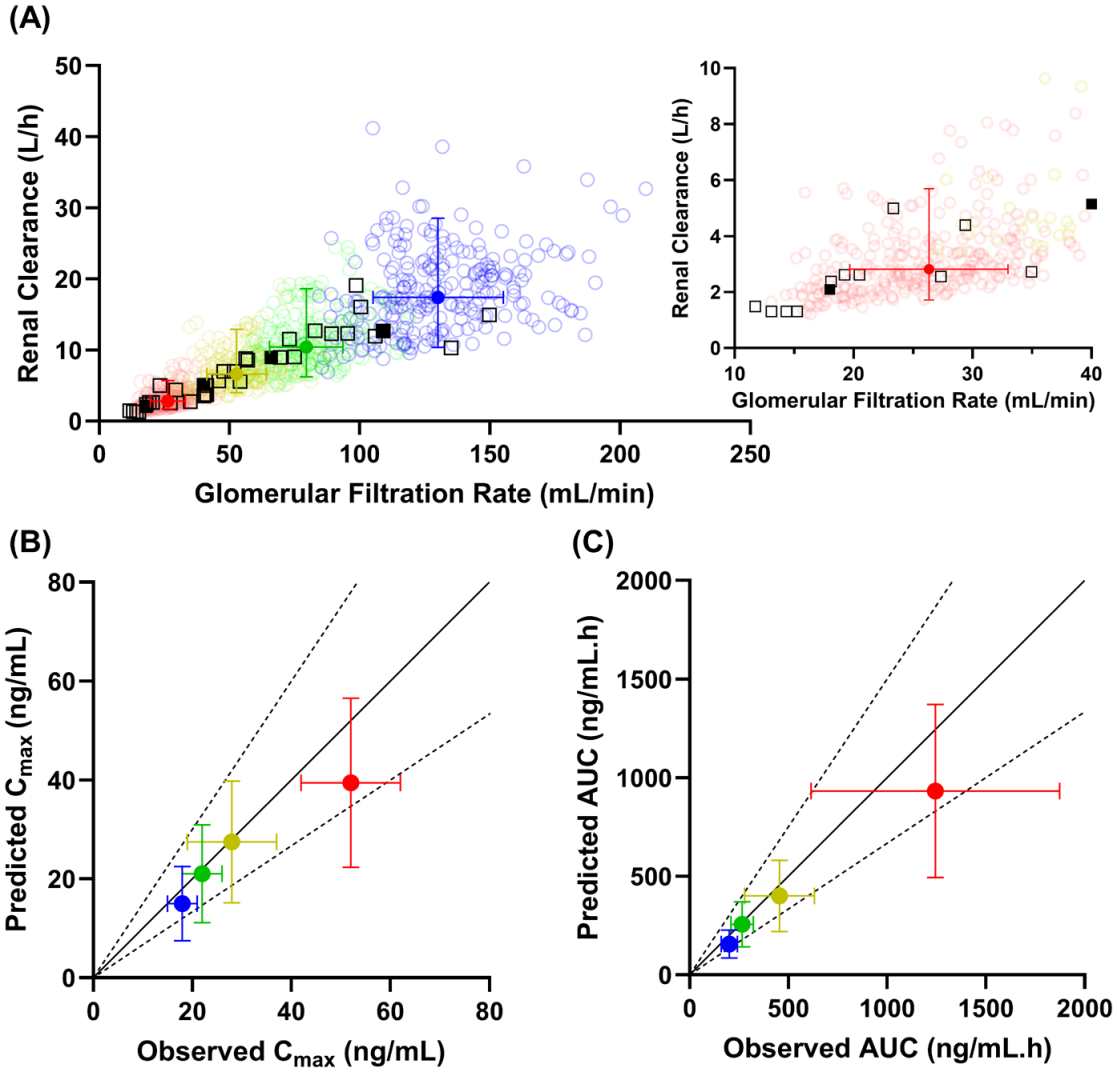
Effect of chronic kidney disease (CKD) on adefovir pharmacokinetics. (A) Relationship between adefovir renal clearance versus glomerular filtration rate. (B) Comparison of predicted versus observed maximum plasma concentration (C_max_) and (C) area under the plasma concentration‐time profile of adefovir in different stages of CKD. Observed adefovir C_max_ and AUC in patients with severe CKD increased by a magnitude of 2.9‐and 6.2‐fold relative to healthy individuals, respectively, which our model was able to predict within 1.1‐fold error (predicted 2.6 and 6.0‐fold increase in C_max_ and AUC, respectively). The solid (error bars) and open symbols represent the mean (standard deviation) and individual pharmacokinetic of each subject. Observed data are represented by the black symbols. Predictions of adefovir PK parameter in healthy individuals, and in patients with mild, moderate and severe CKD are represented by the blue, green, yellow and red symbols, respectively. Simulations were performed using the trial design and demographic data reported in the reference clinical study (Table S4) [26].

The Simcyp renal impaired population files were modified to account for disease‐related changes in the proximal tubular cell number; these decreases were proportional to the simulated decrease in GFR in mild, moderate, and severe CKD. In addition, OAT1 activity was reduced further by 16% and 50% in moderate and severe CKD, respectively (Table S1). These modifications were key to predicting the observed trend of decrease in adefovir CL_r_ as the disease progresses from mild to severe CKD (Figure 4A). The adefovir CKD PBPK model predicted a 6.2‐fold decrease in CL_r_ from 17.37 L/h in healthy individuals to 2.81 L/h in patients with severe CKD, recapitulating the observed 6.1‐fold decrease in CL_r_ (12.67 L/h to 2.09 L/h). Similarly, mean C_max_ and AUC in different stages of CKD were predicted within 1.5‐fold of the observed data (Figure 4B,C).

**FIGURE 5:**
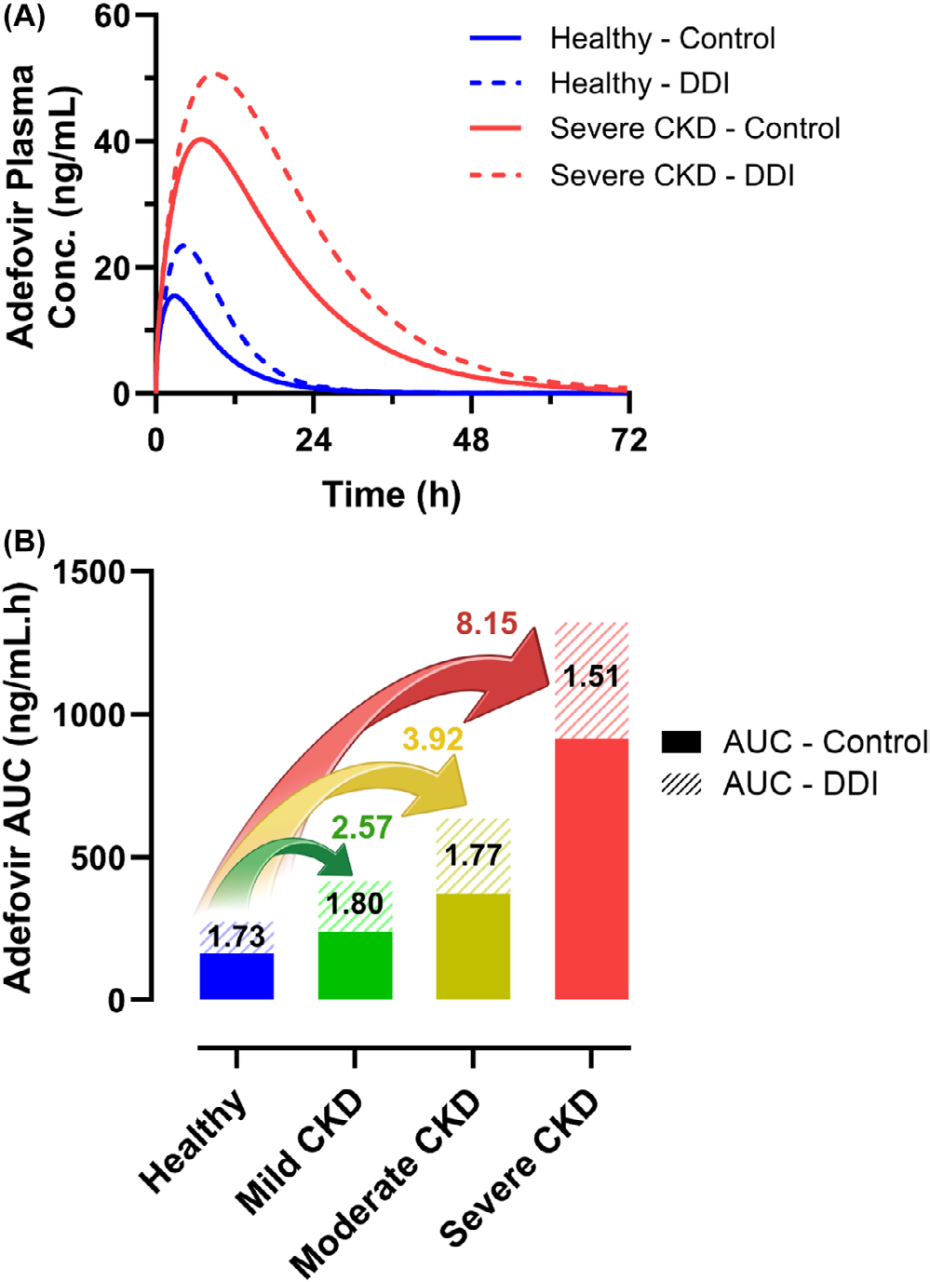
Predicted drug–drug interaction (DDI) between a single oral dose of 10 mg adefovir‐dipivoxil and 1.0 g probenecid in different stages of chronic kidney disease (CKD). (A) Predicted mean adefovir plasma concentration‐time profile in the control and probenecid DDI phase for healthy and severe CKD populations. (B) Predicted impact of probenecid DDI and CKD on adefovir area under the curve (AUC). Solid and dashed bars represent the AUC in the control and DDI phase, respectively. Adefovir AUC ratio due to probenecid inhibition (DDI/control) at each CKD stage is indicated within the dashed bar. The fold increase in AUC due to the combined effect of both DDI and CKD compared against the control phase in healthy individuals is indicated with the colored arrows. Simulations of the combined effect of CKD and probenecid DDI were performed using a generic trial design (Table S5).

Prospective prediction of the DDI between adefovir‐dipivoxil and probenecid revealed a decreasing level of OAT1‐mediated interaction as CKD progresses (Figure 5A). Using a generic simulation trial design (Table S5), the mean adefovir AUCR in the presence of probenecid was predicted to decrease from 1.73 in healthy individuals to 1.51 in severe CKD (Figure 5B). Similarly, the predicted mean C_max_R decreased from 1.52 in healthy individuals to 1.27 in patients with severe CKD. It is important to note that even though adefovir AUCR and C_max_R are predicted to decrease with CKD progression, adefovir exposure is substantially elevated due to both renal impairment and probenecid inhibition when compared against healthy controls. For example, the predicted adefovir AUC in severe CKD and in the absence of any DDI (915 ng/mL.h) was, on average, 5.6‐fold higher than in healthy individuals (162 ng/mL.h) (Figure 5B). This difference in the absolute exposure between healthy individuals and patients with severe CKD increases to 8.2‐fold in the case of the combined effect of disease and probenecid DDI. Likewise, C_max_ is 2.6‐fold higher during severe CKD versus healthy controls, which increases to 3.3‐fold upon the combined probenecid DDI and severe CKD effect.

## 4 Discussion

Collaborative efforts within the drug development community have enabled progress in the prediction of metabolic DDI, creating confidence in the use and subsequent extrapolation of in vitro read‐outs using the PBPK modeling framework [2]. Such concerted effort for the prediction of transporter‐mediated DDI is lagging behind, but may be improved with the introduction of endogenous biomarkers of transporter activity [3, 5, 9]. Combining endogenous biomarker clinical data with PBPK modeling has the potential to inform or even replace the need for dedicated clinical DDI studies with transporter clinical probes. In this work, the DDI between the OAT1 clinical probe (adefovir) and the OAT1/3 clinical probe inhibitor (probenecid) was predicted using PBPK modeling. The adefovir PBPK model was developed using proteomics‐informed IVIVE of OAT1 CL_int_ measured in ciPTEC‐OAT1 [38] and extensively verified with clinical data from different ethnic groups after adefovir and adefovir‐dipivoxil administration. A previously developed and verified probenecid PBPK model [21], incorporating endogenous biomarker‐informed in vivo OAT1/3 K_i_ obtained from PopPK modeling [15] of PDA clinical data [19], was applied to predict the DDI with adefovir. The application of both models successfully predicted the extent of the probenecid‐adefovir interaction after a 0.75 g and 1.5 g single oral dose of probenecid, with a modest overprediction of the DDI with the lowest probenecid dose (0.5 g). AUCR, C_max_R, and CL_r_R were predicted within the stringent Guest criterion for mid‐to‐high doses of probenecid and within 1.5‐fold of observed in all scenarios. Clinical interaction data with weak and moderate OAT1 inhibitors were not available to test the adefovir model further. Nevertheless, the ability of the current adefovir model to predict OAT1 interactions ranging from low to high doses of probenecid is promising and demonstrates the potential of the biomarker‐informed K_i_ concept to be used for such purposes.

Clinical data investigating the DDI between adefovir and the lowest 0.5 g probenecid single oral dose surprisingly did not observe any significant change to adefovir AUC (AUCR = 1.04) [22], in contrast to the 1.45‐fold increase predicted by our PBPK models. Up to 98% of the adefovir dose is recovered in urine after intravenous administration, and active renal secretion contributes approximately 60% to its CL_r_ [25]. The ratio of observed unbound probenecid C_max_ to OAT1/3 K_i_ was 5.1 at a 0.5 g dose; thus, it was expected that adefovir AUC would be sensitive to probenecid inhibition even at this low dose. As this 0.5 g probenecid dose led to an observed 1.2‐ and 1.7‐fold decrease in adefovir CL_r_ and CL_sec_ (calculated by subtracting the product of f_u,p_ and GFR from CL_r_), it is likely that OAT1 inhibition occurred. Subsequent mid‐to‐high doses of 0.75 g and 1.5 g probenecid led to an observed AUCR of 2.1 and 1.8 respectively, which our model correctly predicted. Similarly, the observed 2.3‐fold (0.75 g) and 1.9‐fold (1.5 g) decrease in CL_r_ due to probenecid inhibition was predicted well. The absence of any effect on the reported mean plasma AUC and C_max_ at the lowest probenecid dose may be due to the variable data and small sample size (*N* = 6 subjects) in the clinical study and a relatively large contribution of glomerular filtration to adefovir's total clearance. Adefovir is weakly protein‐bound (f_u,*p*_ = 0.96) and glomerular filtration contributes approximately 40% [25] to its total clearance, limiting its ability to detect weak OAT1 inhibition using only plasma pharmacokinetic data. Clinical data indicated that the effect of maximum OAT1 inhibition on adefovir plasma exposure was relatively modest (AUCR = 1.8–2.1) even with a high dose of probenecid, while the corresponding adefovir CL_sec_ fell below zero, suggesting complete inhibition of OAT1 activity [22]. Tenofovir, another weakly protein‐bound clinical probe of OAT1, showed comparable trends to adefovir (AUCR of 1.6 at high probenecid 2.0 g dose [49]). In contrast, highly protein‐bound OAT1/3 probes with a greater contribution of secretion (e.g., PDA and furosemide) had AUCR > 3 with a probenecid dose of 1.0 g [17]. The dynamic range of DDI effect on AUCR of adefovir and other weakly bound OAT1/3 probes is narrow and may render adefovir insensitive towards detecting weak OAT1 inhibition. Therefore, concurrent monitoring of adefovir and OAT1/3 endogenous biomarker, such as PDA, would enable the assessment of a range of OAT1 inhibitory potencies, as well as the clinical significance of the interaction on highly and weakly protein‐bound OAT1 substrates. Additionally, we recommend that OAT1/3 DDI clinical studies use a probenecid dose greater than 0.5 g single oral dose. While the minimum probenecid dose needed in OAT1/3 DDI clinical studies has not been formally recommended, recent studies have used a total daily dose of at least 1.0 g [13, 17, 19, 20, 49].

Considering above, we further extended our adefovir model to the CKD population, where both glomerular filtration and active secretion are expected to decline. A dedicated clinical study evaluating the impact of renal impairment is recommended in the latest FDA guidance when the fraction of drug eliminated in the urine is equal to or greater than 30% [35]. However, a stand‐alone clinical study in renally impaired populations may not always be feasible, e.g., for oncology drugs [50], thus necessitating alternative approaches to evaluate renal impairment in patients. The extension of the verified adefovir model to renally impaired populations implemented our published recommendations for PBPK modeling of OAT1/3 probes in CKD [27]. Our CKD‐PBPK model was able to recapitulate the changes in adefovir AUC and C_max_ with disease progression (Figure 4), thus further emphasizing the importance of implementing the additional 50% decline in OAT1 activity beyond the deterioration of GFR in severe CKD. The adefovir example builds on our previous modeling efforts of OAT substrates (PDA [21], ganciclovir [45]) increasing the confidence and robustness of PBPK modeling in predicting changes to the pharmacokinetics of OAT drugs in CKD. Investigating the untested scenario of probenecid‐adefovir DDI in CKD populations with our verified PBPK models revealed a trend of declining extent of probenecid inhibitory effect on adefovir plasma exposure (AUCR and C_max_R) with disease progression. However, it needs to be emphasized that plasma AUC of adefovir was still substantially elevated (> 8‐fold greater in severe CKD versus healthy controls) due to the combined effect of the disease and probenecid inhibition. Therefore, co‐administration of both drugs in CKD should remain avoided.

In conclusion, this study has demonstrated the first application of biomarker‐informed PBPK modeling in predicting the probenecid DDI risk with the OAT1 clinical probe adefovir. Further verification of the biomarker‐informed K_i_ concept with inhibitors of varying OAT1 inhibitory potencies and other OAT1 probe substrates is needed. The study also emphasizes the importance of considering the contribution of secretion clearance to total renal clearance when selecting clinical probe substrates of renal transporters. Further application of the adefovir model in patients with renal impairment enabled the accurate prediction of the CKD‐induced changes to adefovir plasma exposure, affirming our recommendation [27] to reduce OAT1 activity beyond the decline of GFR in moderate and severe CKD. Therefore, our study reinforces the role of PBPK modeling in informing transporter‐mediated DDI studies (combined with endogenous biomarker data) and predicting the effect of renal impairment on the pharmacokinetics of OAT1 drugs. Furthermore, additional PBPK modeling of the DDI between adefovir and probenecid models in CKD populations provided insight into the clinical consequences of co‐administering both drugs in renally impaired patients and supports the utility of PBPK modeling of untested scenarios.

## Author Contributions

S.P.F.T., H.W., A.R.‐H., D.S., and A.G. wrote the manuscript. S.P.F.T., D.S., and A.G. designed the research. S.P.F.T., H.W., D.S., and A.G. performed the research. S.P.F.T., H.W., A.R.‐H., D.S., and A.G. analyzed the data. S.P.F.T., H.W., D.S., and A.G. contributed new reagents/analytical tools.

## Conflicts of Interest

A.R.‐H. is an employee and shareholder of Certara Predictive Technologies that produces physiologically based pharmacokinetic modeling tools. All other authors declare no conflicts of interest.

## Supporting information


Data S1.

## References

[1] X. Zhang , Y. Yang , M. Grimstein , et al., “Application of PBPK Modeling and Simulation for Regulatory Decision Making and Its Impact on US Prescribing Information: An Update on the 2018–2019 Submissions to the US FDA's Office of Clinical Pharmacology,” Journal of Clinical Pharmacology 60, no. 1 (2020): S160–S178. 33205429 10.1002/jcph.1767

[2] K. Venkatakrishnan and A. Rostami‐Hodjegan , “Come Dance With Me: Transformative Changes in the Science and Practice of Drug‐Drug Interactions,” Clinical Pharmacology and Therapeutics 105 (2019): 1272–1278. 31004453 10.1002/cpt.1433

[3] K. S. Taskar , V. Pilla Reddy , H. Burt , et al., “Physiologically‐Based Pharmacokinetic Models for Evaluating Membrane Transporter Mediated Drug‐Drug Interactions: Current Capabilities, Case Studies, Future Opportunities, and Recommendations,” Clinical Pharmacology and Therapeutics 107, no. 5 (2020): 1082–1115, 10.1002/cpt.1693. 31628859 PMC7232864

[4] Y. Guo , X. Chu , N. J. Parrott , et al., “Advancing Predictions of Tissue and Intracellular Drug Concentrations Using in Vitro, Imaging and Physiologically Based Pharmacokinetic Modeling Approaches,” Clinical Pharmacology and Therapeutics 104, no. 5 (2018): 865–889, 10.1002/cpt.1183. 30059145 PMC6197917

[5] A. Galetin , K. L. R. Brouwer , D. Tweedie , et al., “Membrane Transporters in Drug Development and as Determinants of Precision Medicine,” Nature Reviews. Drug Discovery 23, no. 4 (2024): 255–280, 10.1038/s41573-023-00877-1. 38267543 PMC11464068

[6] International Council for Harmonisation of Technical Requirements for Pharmaceuticals for Human Use , “ICH Harmonised Guideline on Drug Interaction Studies M12,” 2024, https://database.ich.org/sites/default/files/ICH_M12_Step4_Guideline_2024_0521_0.pdf.

[7] U.S. Food and Drug Administration , “Clinical Drug Interaction Studies—Cytochrome P450 Enzyme‐ and Transporter‐Mediated Drug Interactions: Guidance for Industry,” 2020, https://www.fda.gov/regulatory‐information/search‐fda‐guidance‐documents/clinical‐drug‐interaction‐studies‐cytochrome‐p450‐enzyme‐and‐transporter‐mediated‐drug‐interactions.

[8] European Medicines Agency , “Guideline on the Investigation of Drug Interactions,” 2012, https://www.ema.europa.eu/en/investigation‐drug‐interactions‐scientific‐guideline.

[9] A. D. Rodrigues , “Reimagining the Framework Supporting the Static Analysis of Transporter Drug Interaction Risk; Integrated Use of Biomarkers to Generate Pan‐Transporter Inhibition Signatures,” Clinical Pharmacology and Therapeutics 113 (2023): 986–1002. 35869864 10.1002/cpt.2713

[10] S. C. Lee , V. Arya , X. Yang , D. A. Volpe , and L. Zhang , “Evaluation of Transporters in Drug Development: Current Status and Contemporary Issues,” Advanced Drug Delivery Reviews 116 (2017): 100–118. 28760687 10.1016/j.addr.2017.07.020

[11] N. S. Jones , K. Yoshida , L. Salphati , J. R. Kenny , M. R. Durk , and L. W. Chinn , “Complex DDI by Fenebrutinib and the Use of Transporter Endogenous Biomarkers to Elucidate the Mechanism of DDI,” Clinical Pharmacology and Therapeutics 107 (2020): 269–277. 31376152 10.1002/cpt.1599 PMC6977399

[12] M. Vourvahis , W. Byon , C. Chang , et al., “Evaluation of the Effect of Abrocitinib on Drug Transporters by Integrated Use of Probe Drugs and Endogenous Biomarkers,” Clinical Pharmacology and Therapeutics 112 (2022): 665–675. 35344588 10.1002/cpt.2594 PMC9540496

[13] E. Kimoto , C. Costales , M. A. West , et al., “Biomarker‐Informed Model‐Based Risk Assessment of Organic Anion Transporting Polypeptide 1B Mediated Drug‐Drug Interactions,” Clinical Pharmacology and Therapeutics 111 (2022): 404–415, 10.1002/cpt.2434. 34605015

[14] X. Chu , M. Liao , H. Shen , et al., “Clinical Probes and Endogenous Biomarkers as Substrates for Transporter Drug‐Drug Interaction Evaluation: Perspectives From the International Transporter Consortium,” Clinical Pharmacology and Therapeutics 104 (2018): 836–864. 30347454 10.1002/cpt.1216

[15] A. Ahmad , K. Ogungbenro , A. Kunze , et al., “Population Pharmacokinetic Modeling and Simulation to Support Qualification of Pyridoxic Acid as Endogenous Biomarker of OAT1/3 Renal Transporters,” CPT: Pharmacometrics & Systems Pharmacology 10, no. 5 (2021): 467–477, 10.1002/psp4.12610. 33704919 PMC8129719

[16] S. Barnett , K. Ogungbenro , K. Ménochet , et al., “Gaining Mechanistic Insight Into Coproporphyrin I as Endogenous Biomarker for OATP1B‐Mediated Drug‐Drug Interactions Using Population Pharmacokinetic Modeling and Simulation,” Clinical Pharmacology and Therapeutics 104 (2018): 564–574. 29243231 10.1002/cpt.983 PMC6175062

[17] H. Shen , V. K. Holenarsipur , T. T. Mariappan , et al., “Evidence for the Validity of Pyridoxic Acid (PDA) as a Plasma‐Based Endogenous Probe for OAT1 and OAT3 Function in Healthy Subjects,” Journal of Pharmacology and Experimental Therapeutics 368, no. 1 (2019): 136–145, 10.1124/jpet.118.252643. 30361237

[18] H. Shen , D. M. Nelson , R. V. Oliveira , et al., “Discovery and Validation of Pyridoxic Acid and Homovanillic Acid as Novel Endogenous Plasma Biomarkers of Organic Anion Transporter (OAT) 1 and OAT3 in Cynomolgus Monkeys,” Drug Metabolism and Disposition 46 (2018): 178–188. 29162614 10.1124/dmd.117.077586

[19] M. E. Willemin , T. K. van der Made , I. Pijpers , et al., “Clinical Investigation on Endogenous Biomarkers to Predict Strong OAT‐Mediated Drug‐Drug Interactions,” Clinical Pharmacokinetics 60, no. 9 (2021): 1187–1199, 10.1007/s40262-021-01004-2. 33840062

[20] A. Gessner , F. Müller , P. Wenisch , et al., “A Metabolomic Analysis of Sensitivity and Specificity of 23 Previously Proposed Biomarkers for Renal Transporter‐Mediated Drug‐Drug Interactions,” Clinical Pharmacology and Therapeutics 114 (2023): 1058–1072. 37540045 10.1002/cpt.3017

[21] S. P. F. Tan , M. E. Willemin , J. Snoeys , et al., “Development of 4‐Pyridoxic Acid PBPK Model to Support Biomarker‐Informed Evaluation of OAT1/3 Inhibition and Effect of Chronic Kidney Disease,” Clinical Pharmacology and Therapeutics 114 (2023): 1243–1253. 37620246 10.1002/cpt.3029

[22] K. Maeda , Y. Tian , T. Fujita , et al., “Inhibitory Effects of p‐Aminohippurate and Probenecid on the Renal Clearance of Adefovir and Benzylpenicillin as Probe Drugs for Organic Anion Transporter (OAT) 1 and OAT3 in Humans,” European Journal of Pharmaceutical Sciences 59 (2014): 94–103, 10.1016/j.ejps.2014.04.004. 24747579

[23] U.S. Food and Drug Administration , “CYP Enzyme‐ and Transporter System‐Based Clinical Substrates, Inhibitors, or Inducers,” 2023, accessed January 6 2024, https://www.fda.gov/drugs/drug‐interactions‐labeling/healthcare‐professionals‐fdas‐examples‐drugs‐interact‐cyp‐enzymes‐and‐transporter‐systems.

[24] C. Trueck , C. H. Hsin , O. Scherf‐Clavel , et al., “A Clinical Drug‐Drug Interaction Study Assessing a Novel Drug Transporter Phenotyping Cocktail With Adefovir, Sitagliptin, Metformin, Pitavastatin, and Digoxin,” Clinical Pharmacology and Therapeutics 106, no. 6 (2019): 1398–1407, 10.1002/cpt.1564. 31247117

[25] K. C. Cundy , P. Barditch‐Crovo , R. E. Walker , et al., “Clinical Pharmacokinetics of Adefovir in Human Immunodeficiency Virus Type 1‐Infected Patients,” Antimicrobial Agents and Chemotherapy 39, no. 11 (1995): 2401–2405, 10.1128/AAC.39.11.2401. 8585716 PMC162955

[26] U.S. Food and Drug Administration , “Hepsera Clinical Pharmacology and Biopharmaceutics Review,” *NDA: 21–449* 2002.

[27] S. P. F. Tan , D. Scotcher , A. Rostami‐Hodjegan , and A. Galetin , “Effect of Chronic Kidney Disease on the Renal Secretion via Organic Anion Transporters 1/3: Implications for Physiologically‐Based Pharmacokinetic Modeling and Dose Adjustment,” Clinical Pharmacology and Therapeutics 112 (2022): 643–652. 35569107 10.1002/cpt.2642 PMC9540491

[28] C. H. Hsueh , K. Yoshida , P. Zhao , et al., “Identification and Quantitative Assessment of Uremic Solutes as Inhibitors of Renal Organic Anion Transporters, OAT1 and OAT3,” Molecular Pharmaceutics 13 (2016): 3130–3140. 27467266 10.1021/acs.molpharmaceut.6b00332

[29] A. Chapron , D. D. Shen , B. R. Kestenbaum , C. Robinson‐Cohen , J. Himmelfarb , and C. K. Yeung , “Does Secretory Clearance Follow Glomerular Filtration Rate in Chronic Kidney Diseases? Reconsidering the Intact Nephron Hypothesis,” Clinical and Translational Science 10 (2017): 395–403. 28675584 10.1111/cts.12481 PMC5593164

[30] B. P. Kearney , S. Ramanathan , A. K. Cheng , R. Ebrahimi , and J. Shah , “Systemic and Renal Pharmacokinetics of Adefovir and Tenofovir Upon Coadministration,” Journal of Clinical Pharmacology 45 (2005): 935–940. 16027404 10.1177/0091270005278949

[31] Y. Shida , S. Nohda , A. S. Gross , J. L. Palmer , K. Morimoto , and A. Egawa , “Pharmacokinetics of Adefovir After Oral Administration of Adefovir Dipivoxil 10 Mg in Healthy Japanese Males and Japanese Patients With Chronic Hepatitis B,” Rinsho Yakuri/Japanese Journal of Clinical Pharmacology and Therapeutics 36 (2005): 289–296.

[32] D. Q. Sun , H. S. Wang , M. Y. Ni , B. J. Wang , and R. C. Guo , “Pharmacokinetics, Safety and Tolerance of Single‐ and Multiple‐Dose Adefovir Dipivoxil in Healthy Chinese Subjects,” British Journal of Clinical Pharmacology 63 (2007): 15–23. 16869815 10.1111/j.1365-2125.2006.02728.x PMC2000720

[33] B. S. Fok , S. Gardner , S. Piscitelli , et al., “Pharmacokinetic Properties of Single‐Dose Lamivudine/Adefovir Dipivoxil Fixed‐Dose Combination in Healthy Chinese Male Volunteers,” Clinical Therapeutics 35 (2013): 68–76. 23274144 10.1016/j.clinthera.2012.12.001

[34] P. Barditch‐Crovo , J. Toole , C. W. Hendrix , et al., “Anti‐Human Immunodeficiency Virus (HIV) Activity, Safety, and Pharmacokinetics of Adefovir Dipivoxil (9‐[2‐(Bis‐Pivaloyloxymethyl)‐phosphonylmethoxyethyl]Adenine) in HIV‐Infected Patients,” Journal of Infectious Diseases 176, no. 2 (1997): 406–413, 10.1086/514057. 9237705

[35] U.S. Food and Drug Administration , “Pharmacokinetics in Patients with Impaired Renal Function – Study Design, Data Analysis, and Impact on Dosing: Guidance for Industry,” 2024, https://www.fda.gov/regulatory‐information/search‐fda‐guidance‐documents/pharmacokinetics‐patients‐impaired‐renal‐function‐study‐design‐data‐analysis‐and‐impact‐dosing.

[36] T. Rodgers , D. Leahy , and M. Rowland , “Physiologically Based Pharmacokinetic Modeling 1: Predicting the Tissue Distribution of Moderate‐To‐Strong Bases,” Journal of Pharmaceutical Sciences 94 (2005): 1259–1276. 15858854 10.1002/jps.20322

[37] T. Rodgers and M. Rowland , “Physiologically Based Pharmacokinetic Modelling 2: Predicting the Tissue Distribution of Acids, Very Weak Bases, Neutrals and Zwitterions,” Journal of Pharmaceutical Sciences 95 (2006): 1238–1257. 16639716 10.1002/jps.20502

[38] S. P. F. Tan , A. Tillmann , S. J. Murby , A. Rostami‐Hodjegan , D. Scotcher , and A. Galetin , “Albumin‐Mediated Drug Uptake by Organic Anion Transporter 1/3 Is Real: Implications for the Prediction of Active Renal Secretion Clearance,” Molecular Pharmaceutics 21 (2024): 4603–4617. 39166754 10.1021/acs.molpharmaceut.4c00504 PMC11372837

[39] Z. M. Al‐Majdoub , D. Scotcher , B. Achour , J. Barber , A. Galetin , and A. Rostami‐Hodjegan , “Quantitative Proteomic Map of Enzymes and Transporters in the Human Kidney: Stepping Closer to Mechanistic Kidney Models to Define Local Kinetics,” Clinical Pharmacology and Therapeutics 110 (2021): 1389–1400. 34390491 10.1002/cpt.2396

[40] T. Imaoka , H. Kusuhara , M. Adachi , J. D. Schuetz , K. Takeuchi , and Y. Sugiyama , “Functional Involvement of Multidrug Resistance‐Associated Protein 4 (MRP4/ABCC4) in the Renal Elimination of the Antiviral Drugs Adefovir and Tenofovir,” Molecular Pharmacology 71 (2007): 619–627. 17110501 10.1124/mol.106.028233

[41] T. Fujita , C. Brown , E. J. Carlson , et al., “Functional Analysis of Polymorphisms in the Organic Anion Transporter, SLC22A6 (OAT1),” Pharmacogenetics and Genomics 15, no. 4 (2005): 201–209, 10.1097/01213011-200504000-00003. 15864112

[42] E. J. Guest , L. Aarons , J. B. Houston , A. Rostami‐Hodjegan , and A. Galetin , “Critique of the Two‐Fold Measure of Prediction Success for Ratios: Application for the Assessment of Drug‐Drug Interactions,” Drug Metabolism and Disposition 39 (2011): 170–173. 21036951 10.1124/dmd.110.036103

[43] N. S. Bricker , “On the Meaning of the Intact Nephron Hypothesis,” American Journal of Medicine 46 (1969): 1–11. 4952757 10.1016/0002-9343(69)90053-9

[44] D. Scotcher , C. R. Jones , A. Galetin , and A. Rostami‐Hodjegan , “Delineating the Role of Various Factors in Renal Disposition of Digoxin Through Application of Physiologically Based Kidney Model to Renal Impairment Populations,” Journal of Pharmacology and Experimental Therapeutics 360 (2017): 484–495. 28057840 10.1124/jpet.116.237438 PMC5370399

[45] D. Scotcher and A. Galetin , “PBPK Simulation‐Based Evaluation of Ganciclovir Crystalluria Risk Factors: Effect of Renal Impairment, Old Age, and Low Fluid Intake,” AAPS Journal 24, no. 13 (2021): 1–17. 34907479 10.1208/s12248-021-00654-1 PMC8816528

[46] Lannett Company, I , “Benemid (Probenecid) Package Insert,” 1976.

[47] Gilead Sciences, I , “Hepsera (Adefovir‐Dipivoxil) Product Label,” *Reference ID: 3094424* 2012.

[48] C. H. Hsueh , V. Hsu , P. Zhao , L. Zhang , K. M. Giacomini , and S. M. Huang , “PBPK Modeling of the Effect of Reduced Kidney Function on the Pharmacokinetics of Drugs Excreted Renally by Organic Anion Transporters,” Clinical Pharmacology and Therapeutics 103 (2018): 485–492. 28738449 10.1002/cpt.750

[49] S. N. Liu , B. T. Gufford , J. B. L. Lu , et al., “Inhibitory Effects of Probenecid on Pharmacokinetics of Tenofovir Disoproxil Fumarate and Emtricitabine for on‐Demand HIV Preexposure Prophylaxis,” Clinical Pharmacology and Therapeutics 107, no. 5 (2020): 1200–1208, 10.1002/cpt.1714. 31675437 PMC7703849

[50] P. Ravenstijn , M. Chetty , and P. Manchandani , “Design and Conduct Considerations for Studies in Patients With Impaired Renal Function,” Clinical and Translational Science 14 (2021): 1689–1704. 33982447 10.1111/cts.13061 PMC8504825

