# Supplementary material for: Evaluation of Adefovir PBPK Model to Assess Biomarker‐Informed OAT1 Drug–Drug Interaction and Effect of Chronic Kidney Disease
Source: CPT Pharmacometrics Syst Pharmacol. 2025 Mar 3;14(5):964–74. doi: 10.1002/psp4.70010 (PMC12072229; doi:10.1002/psp4.70010)
Supplement: Supplementary file 1 — Data S1. [file PSP4-14-964-s001.docx]

**Supplementary Material**

Evaluation of Adefovir PBPK Model to Assess Biomarker-Informed OAT1 Drug-Drug Interaction and Effect of Chronic Kidney Disease

Shawn Pei Feng Tan^1^, Huaying Wu^1^, Amin Rostami-Hodjegan^1,2^, Daniel Scotcher^1^ and Aleksandra Galetin^1^

^1^ Centre for Applied Pharmacokinetic Research, School of Heath Sciences, University of Manchester, Manchester, UK

^2^ Certara Predictive Technologies, Sheffield, UK

Current affiliation for H. Wu: Drug Metabolism and Pharmacokinetics Department, Sygnature Discovery Ltd, BioCity, Nottingham, UK.

SUPPLEMENTARY FIGURES

| **(A)** | **(B)** |
| --- | --- |
| 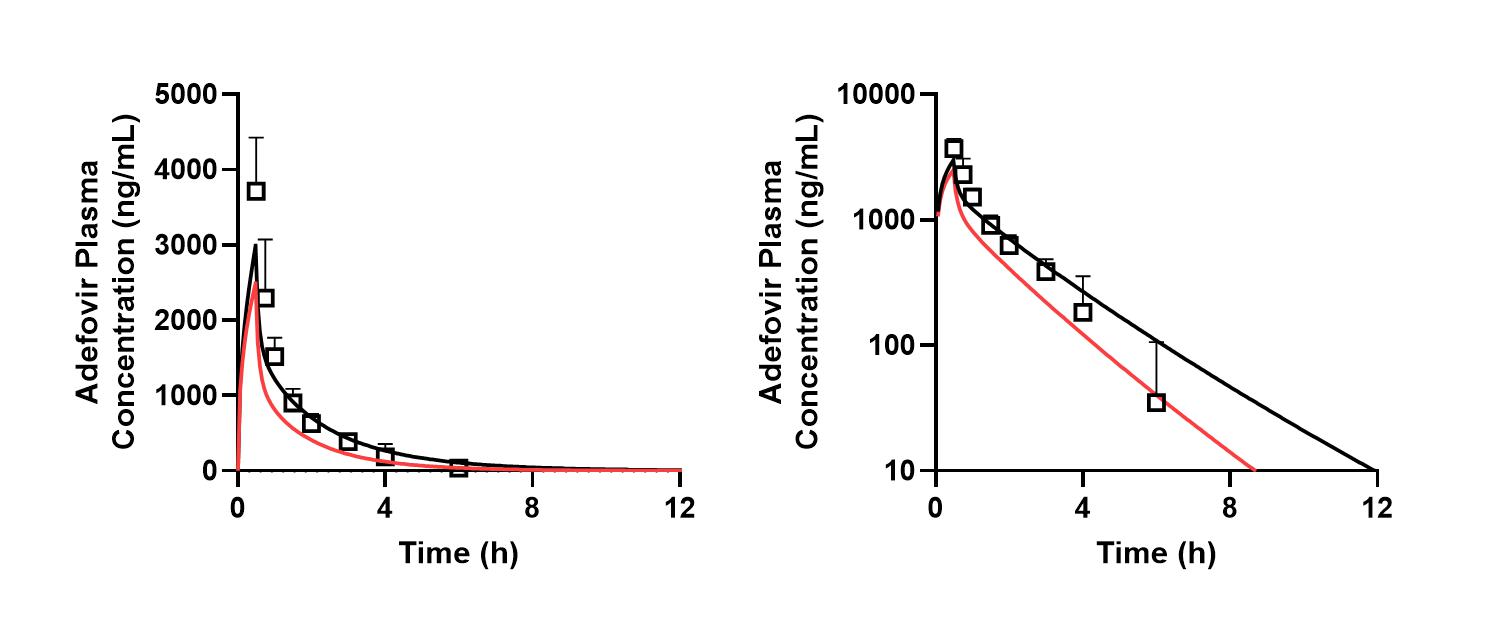 | |
| **Figure S1.** Adefovir plasma concentration-time profile in linear **(A)** and log scale **(B)** after intravenous administration of adefovir 1.0mg/kg. The red and black line represents the predicted mean plasma concentration-time profile using the measured relative expression factor (REF) of 8.0^1^ or the optimised REF of 2.0, respectively. Symbols (error bars) represent the observed data from Cundy *et al*.^2^ (mean ± standard deviation). | |

| **(A)** | **(B)** |
| --- | --- |
| 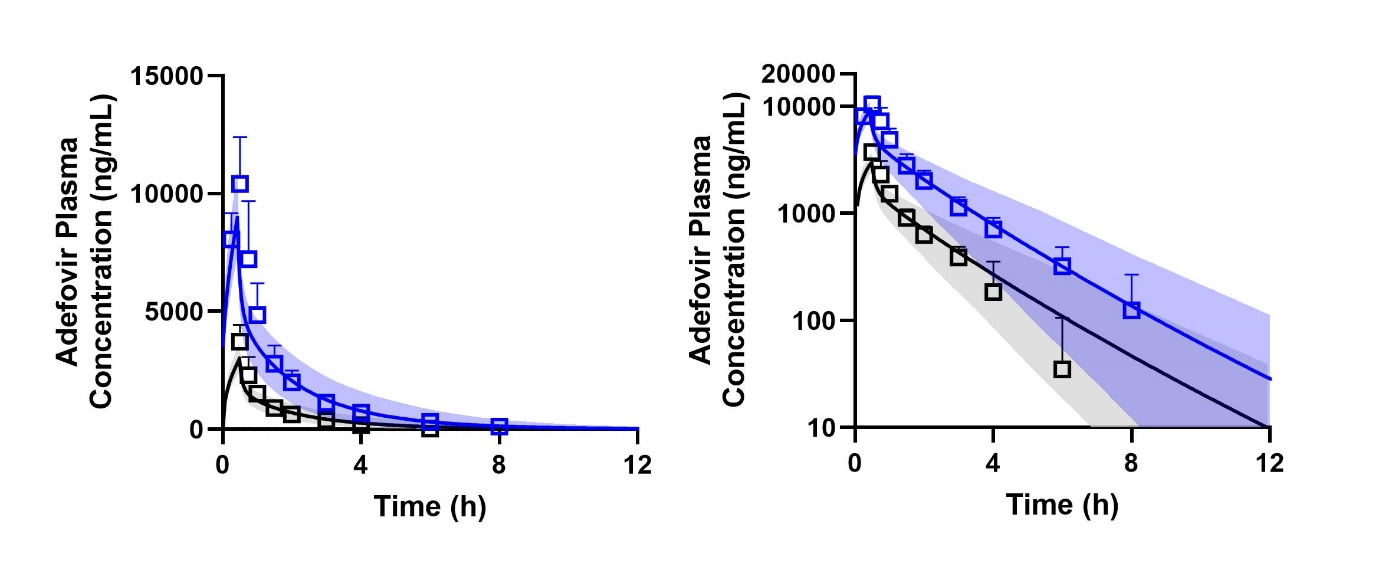 | |
| **Figure S2.** Adefovir plasma concentration-time profile in linear **(A)** and log scale **(B)** after intravenous administration of adefovir 1.0g/kg (black line/symbols) and 3.0mg/kg (blue line/symbols). Predicted mean and 5^th^ to 95^th^ percentile is represented by the solid line and shaded area respectively. Symbols (error bars) represent the observed data from Cundy *et al*.^2^ (mean ± standard deviation). | |

| 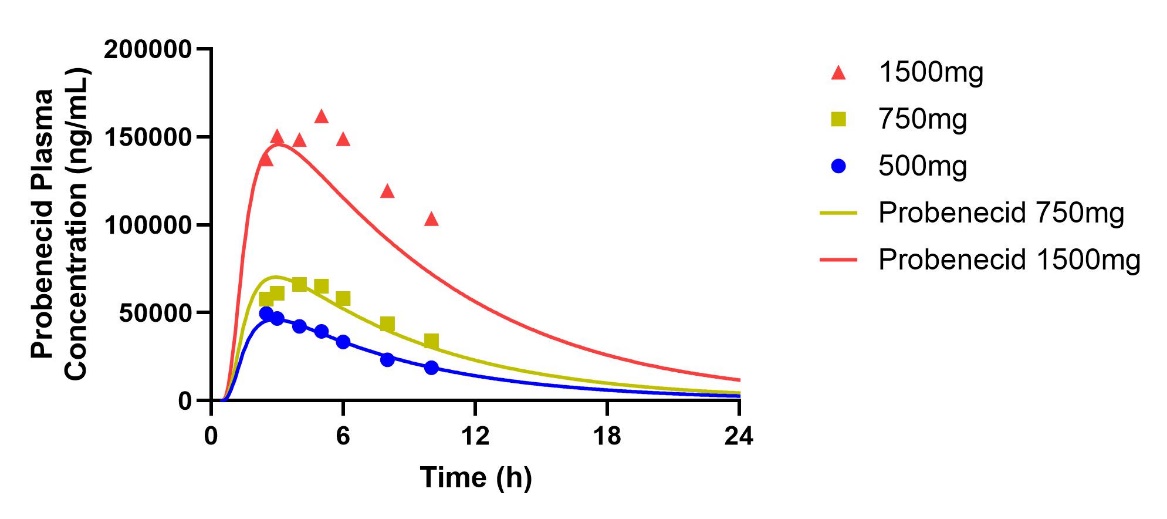 |
| --- |
| **Figure S3.** Predicted versus observed probenecid plasma concentration versus time profile after 0.5g (blue), 0.75g (yellow) and 1.5g (red) single oral dose of probenecid. The coloured symbols and solid line represent the observed mean plasma concentration from Maeda *et al*.^3^ and model predictions respectively. |

| 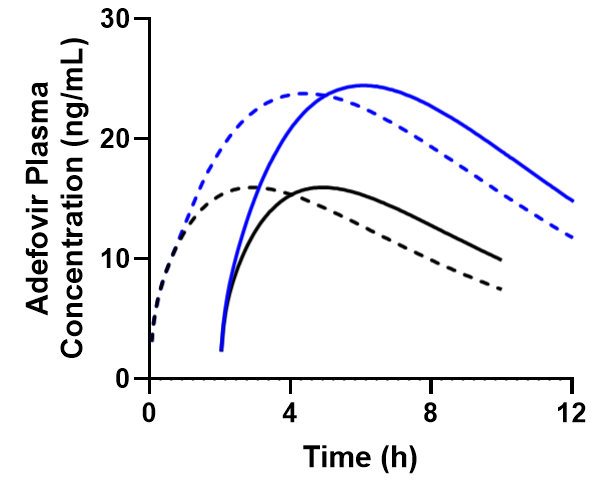 |
| --- |
| **Figure S4.** Predicted adefovir plasma concentration-time profile during the DDI with 1.0g single oral dose of probenecid administered simultaneously (dashed lines) or two hours prior (solid lines) to 10 mg single oral dose of adefovir-dipivoxil. Predicted mean adefovir plasma concentration in the control and DDI phase is represented by the black and blue lines, respectively. |

SUPPLEMENTARY TABLES

**Table S1.** PBPK model input parameters for adefovir in healthy and modifications to systems parameters for chronic kidney disease simulations

| **Model Input Parameters** | **Value [%CV]** | **Reference** |
| --- | --- | --- |
| Molecular weight (g/mol) | 273.19 |  |
| LogP | -1.8 | Predicted using ChemAxon^4^ |
| Compound type | Diprotic Acid |  |
| pKa | 2.0  6.8 | Measured^2^ |
| B/P ratio | 0.55 | Assumption |
| Fraction unbound in plasma | 0.96 | Measured^5^ |
| **Absorption** | | |
| Absorption Model | 1^st^ Order |  |
| f_a_ | 0.47 [30%] | Assumption^6^, within range of reported estimated bioavailability (0.32 to 0.59^5,7^) |
| k_a_ (1/h) | 0.174 [30%] | Parameter estimate using clinical data after oral administration of adefovir-dipivoxil^8^ |
| **Distribution and Elimination** | | |
| Distribution model | Full PBPK Model |  |
| Prediction method | Method 2 | Rodgers and Rowland method^9,10^ |
| V_ss_ (L/kg) | 0.39 |  |
| **Mechanistic Kidney Model** | | |
| CL_pd_ (µL/min/10^6^ PTC) | 0.017 | Measured experimentally^1^ |
| OAT1 CL_int_ (µL/min/10^6^ PTC) | 5.34 |  |
| OAT1 RAF/REF | 2.0 | Optimised using adefovir clinical data after intravenous administration^2^ |
| MRP4 CL_int_ (µL/min/10^6^ PTC) | 1.0 | Assuming OAT1 uptake is the rate-limiting process of adefovir renal secretion |
| MRP4 RAF/REF | 1 |  |
| f_u,kidney-cell_ | 1 |  |
| **Additional Modifications to Simcyp Renal Impaired Population File** | | |
| OAT1 Relative Abundance | 1.0 (Mild CKD)  0.84 (Moderate CKD)  0.50 (Severe CKD) | Estimated^11^, details in Methods section |
| PTC per gram of kidney cortex  (10^6^ PTC/g kidney) | 55.7 (Mild CKD)  51.8 (Moderate CKD)  31.8 (Severe CKD) | Declines proportionally to GFR in CKD as done previously^12,13^, details in Methods section. |

B/P, blood to plasma ratio; CL_int_, intrinsic clearance; CKD, chronic kidney disease; CL_pd_, passive transport clearance from the basolateral-apical and apical-basolateral direction; f_a_, fraction absorbed; f_u,kidney cell_, fraction unbound in the renal cell ; IV, intravenous ; k_a_, absorption rate constant; MRP4, multidrug resistance-associated protein 4; OAT1, organic anion transporter 1; pK_a_, acid dissociation constant; PTC, proximal tubular cells; RAF/REF, relative activity/expression factor, V_ss_, volume of distribution at steady-state

**Table S2.** Model input parameters of the previously verified probenecid PBPK model.^12^

| **Model Input Parameters** | **Value** |
| --- | --- |
| Molecular weight (g/mol) | 285.36 |
| LogP | 3.21 |
| Compound type | Monoprotic Acid |
| pKa | 3.40 |
| B/P ratio | 0.55 |
| Fraction unbound in plasma | 0.100 |
| Absorption Model | ADAM |
| P_eff,man_ (10^-4^ cm/s) | 1.73 |
| f_a_ | 0.899 |
| k_a_ (1/h) | 0.755 |
| PSA (Å) | 85.24 |
| f_u,gut_ | 1 |
| Distribution model | Full PBPK Model |
| Prediction method | Method 2 |
| V_ss_ (L/kg) | 0.111 |
| HLM V_max_ (pmol/min/mg protein) | 261.8 |
| HLM K_m_ (µM) | 76.8 |
| Active Hepatic Scalar (Net) | 1 |
| CL_r_ (L/h) | 0.09 |
| OAT1/3 K_i,unbound_ (µM) | 3.4 |

B/P, blood to plasma ratio; CL_r_, renal clearance; f_a_, fraction absorbed; f_u,gut_, fraction unbound in the enterocytes; HLM, human liver microsome; k­_a_, absorption rate constant; K_i,_ unbound inhibition constant; K_m_, Michaelis–Menten constant; K_p_, tissue:plasma partition coefficient; OAT1/3, Organic Anion Transporter 1/3; P_eff,man_, Effective human jejunum permeability; pK_a_, acid dissociation constant; PSA, polar surface area; Q_gut_, flow rate for overall delivery of drug to the gut; V_max_, maximal metabolic rate, V_ss_, volume of distribution at steady-state;

**Table S3.** Simulation trial design for adefovir and probenecid drug-drug interaction

|  | **Simulation Trial Design** |
| --- | --- |
| **Adefovir-Dipivoxil Dose** | 10mg Single Oral Dose |
| **Probenecid Dose** | 0.5g, 0.75g or 1.5g Single Oral Dose given two hours before adefovir-dipivoxil |
| **Age Range** | 20-31 |
| **No. of Trials** | 40 trials |
| **No. of Subjects** | 6 subjects |
| **Female proportion** | 0% |

**Table S4.** Simulation trial design for adefovir chronic kidney disease (CKD) application

|  | **Simulation Trial Design** | | | |
| --- | --- | --- | --- | --- |
| **CKD Stage** | **Healthy** | **Mild** | **Moderate** | **Severe** |
| **Adefovir-Dipivoxil Dose** | 10mg Single Oral Dose | | | |
| **Age Range** | 21-65 | 40-70 | 38-71 | 36-70 |
| **No. of Trials** | 30 trials | | | |
| **No. of Subjects** | 8 | 8 | 7 | 10 |
| **Female proportion** | 25% | 63% | 57% | 10% |

**Table S5.** Simulation trial design to predict the untested scenario of adefovir and probenecid drug-drug interaction in chronic kidney disease populations

|  | **Simulation Trial Design** |
| --- | --- |
| **Adefovir-Dipivoxil Dose** | 10mg Single Oral Dose |
| **Probenecid Dose** | 1.0g Single Oral Dose |
| **Age Range** | 20-65 |
| **No. of Trials** | 20 trials |
| **No. of Subjects** | 10 subjects |
| **Female proportion** | 50% |

REFERENCES

(1) Tan, S.P.F., Tillmann, A., Murby, S.J., Rostami-Hodjegan, A., Scotcher, D. & Galetin, A. Albumin-Mediated Drug Uptake by Organic Anion Transporter 1/3 Is Real: Implications for the Prediction of Active Renal Secretion Clearance. *Mol Pharm* **21**, 4603-17 (2024).

(2) Cundy, K.C. *et al.* Clinical pharmacokinetics of adefovir in human immunodeficiency virus type 1-infected patients. *Antimicrob Agents Chemother* **39**, 2401-5 (1995).

(3) Maeda, K. *et al.* Inhibitory effects of p-aminohippurate and probenecid on the renal clearance of adefovir and benzylpenicillin as probe drugs for organic anion transporter (OAT) 1 and OAT3 in humans. *Eur J Pharm Sci* **59**, 94-103 (2014).

(4) *FoodDB Version 1.0*. <<www.foodb.ca>>. Accessed 5 August 2022.

(5) U.S. Food and Drug Administration. Hepsera Clinical Pharmacology and Biopharmaceutics Review. *NDA: 21-449*, (2002).

(6) Hsueh, C.H., Hsu, V., Zhao, P., Zhang, L., Giacomini, K.M. & Huang, S.M. PBPK Modeling of the Effect of Reduced Kidney Function on the Pharmacokinetics of Drugs Excreted Renally by Organic Anion Transporters. *Clin Pharmacol Ther* **103**, 485-92 (2018).

(7) Barditch-Crovo, P. *et al.* Anti-human immunodeficiency virus (HIV) activity, safety, and pharmacokinetics of adefovir dipivoxil (9-[2-(bis-pivaloyloxymethyl)-phosphonylmethoxyethyl]adenine) in HIV-infected patients. *J Infect Dis* **176**, 406-13 (1997).

(8) Trueck, C. *et al.* A Clinical Drug-Drug Interaction Study Assessing a Novel Drug Transporter Phenotyping Cocktail With Adefovir, Sitagliptin, Metformin, Pitavastatin, and Digoxin. *Clin Pharmacol Ther* **106**, 1398-407 (2019).

(9) Rodgers, T. & Rowland, M. Physiologically based pharmacokinetic modelling 2: predicting the tissue distribution of acids, very weak bases, neutrals and zwitterions. *J Pharm Sci* **95**, 1238-57 (2006).

(10) Rodgers, T., Leahy, D. & Rowland, M. Physiologically based pharmacokinetic modeling 1: predicting the tissue distribution of moderate-to-strong bases. *J Pharm Sci* **94**, 1259-76 (2005).

(11) Tan, S.P.F., Scotcher, D., Rostami-Hodjegan, A. & Galetin, A. Effect of Chronic Kidney Disease on the Renal Secretion via Organic Anion Transporters 1/3: Implications for Physiologically-Based Pharmacokinetic Modeling and Dose Adjustment. *Clin Pharmacol Ther* **112**, 643-52 (2022).

(12) Tan, S.P.F. *et al.* Development of 4-Pyridoxic Acid PBPK Model to Support Biomarker-Informed Evaluation of OAT1/3 Inhibition and Effect of Chronic Kidney Disease. *Clin Pharmacol Ther* **114**, 1243-53 (2023).

(13) Scotcher, D. & Galetin, A. PBPK Simulation-Based Evaluation of Ganciclovir Crystalluria Risk Factors: Effect of Renal Impairment, Old Age, and Low Fluid Intake. *AAPS J* **24**, 13 (2021).
